# The Adsorption of Methylene Blue by an Amphiphilic Block Co-Poly(Arylene Ether Nitrile) Microsphere-Based Adsorbent: Kinetic, Isotherm, Thermodynamic and Mechanistic Studies

**DOI:** 10.3390/nano9101356

**Published:** 2019-09-21

**Authors:** Xuefei Zhou, Mingzhen Xu, Lingling Wang, Xiaobo Liu

**Affiliations:** Research Branch of Advanced Functional Materials, School of Materials and Energy, University of Electronic Science and Technology of China, Chengdu 611731, China; zhouxuefei0@hotmail.com (X.Z.); wangll@std.uestc.edu.cn (L.W.)

**Keywords:** poly(arylene ether nitrile), microspheres, methylene blue, adsorption

## Abstract

Dye pollution is a serious problem in modern society. We desired to develop an efficient adsorbent for the decontamination of discharged dyes. In this work, the polymeric microspheres derived from a kind of amphiphilic block of co-poly(arylene ether nitrile) (B-*b*-S-P) were prepared on the basis of “oil-in-water” (O/W) microemulsion method. The B-*b*-S-P microspheres were found competent to remove the cationic dye, methylene blue (MB); and various influential factors, such as contact time, initial concentration, solution pH and temperature were investigated. Results indicated that the maximum adsorption capacity of B-*b*-S-P microspheres for MB was 119.84 mg/g at 25 °C in neutral conditions. Adsorption kinetics and isotherm dates were well fitted to a pseudo-second-order kinetic model and the Langmuir isotherm model, and thermodynamic parameters implied that the adsorption process was endothermic. The B-*b*-S-P microspheres also exhibited a highly selective adsorption for cationic dye MB, even in the presence of anionic dye methyl orange (MO). In addition, the possible adsorption mechanism was studied, suggesting that the electrostatic interaction and π–π interaction could be the main force in the adsorption process.

## 1. Introduction

Nowadays, the advances of technology have stimulated versatile dyes applications, and an increasing number of dye-related textile, leather, paper-making, printing and food factories have been developed to meet people’s demands [1,2,3]. However, the affiliated dyes effluents are carcinogenic and non-biodegradable, which could largely damage the ecological balance and human health when they are discharged without rational disposal [4,5]. Numerous strategies have been proposed to relieve the dyes’ pollution pressure in the last few decades, including adsorption, photocatalysis, chemical coagulation/flocculation, microbial degradation, membrane filtration, etc. [6,7,8,9,10]. Among them, adsorption has been regarded as an effective method for the treatment of dye-wastewater, owing to its high efficiency, easy operation, low cost and absence of secondary pollution [11]. In particular, the micro-/nanostructured polymers distinguished themselves as promising adsorbents. Benefiting from the tunable sizes, functional groups and morphologies, polymeric adsorbents displayed advantages when removing dyes based on electrostatic interactions, π–π stacking, hydrophobic interactions and so on [12,13]. For example, Fu et al. prepared the polydopamine microspheres by an oxidative polymerization method, and the microspheres exhibited selective adsorption toward cationic dyes in aqueous solution [14].

To date, the adsorbent derived from an amphiphilic block copolymer has aroused intensive interests [15]. Originating from the self-assembly of a small-molecular surfactant, amphiphilic block copolymer has been exploited to prepare polymeric core-shell structures, such as spherical micelles, vesicles, rods, lamellae and so on [16]. An “oil-in-water” (O/W) microemulsion method has been successfully exploited to fabricate polymeric aggregates, whose morphologies were highly dependent on the solvent in the system [17]. Due to the selective solubility of hydrophobic and hydrophilic blocks in solvents (oil phase and water phase), the amphiphilic block polymer was likely to form a micro-aggregate with the hydrophilic corona and hydrophobic core [18,19]. Taking polystyrene-*b*-poly(acrylic acid) (PS-*b*-PAA) copolymer as example, the PS_200_-*b*-PAA_18_ tended to self-assemble into spheres when the common solvent was DMF, but yield large compound micelles in THF [20]. Therefore, the amphiphilic block copolymer should be promising for fabricating functional micro/nanostructures in suitable conditions.

Poly(arylene ether nitrile) (PEN) is a typical thermoplastic polymer with an aromatic backbone, which is known for its excellent mechanical properties and thermal stability [21,22]. Owing to an abundant source of aromatic diphenol and dihalobenzonitrile, a series of target PENs have been successfully synthesized and functionalized [23,24,25]. Though the aromatic backbone of PEN was inherently hydrophobic, tunable synthesis route and versatile functional groups have made amphiphilic PENs accessible [26]. For instance, a kind of sulfonated poly(arylene ether nitrile) (SPEN) adsorbent has been prepared with pendent sulfonate and carboxylate groups on the side chains, which presented an overall high water-absorption capacity in aqueous solution. Moreover, these hydrophilic groups have been certificated to be crucial for removing cationic dyes in our previous work [27].

Herein, we successfully synthesized a kind of amphiphilic block co-poly(arylene ether nitrile) (B-*b*-S-P), which was capable of self-assembling into polymeric microspheres via an O/W microemulsion method. The prepared B-*b*-S-P microspheres exhibited excellent adsorption performance with MB in an aqueous solution. Moreover, the effect of the dye’s concentration, contact time and solution pH were systematically investigated to reveal the adsorption kinetics, isotherm and mechanism of MB’s adsorption by B-*b*-S-P microspheres. A series of experiments suggested the functional groups and conjugated structure of B-*b*-S-P microspheres were crucial for the adsorption of MB, suggesting the B-*b*-S-P microspheres have potential for disposing of contaminant dyes.

## 2. Materials and Methods

### 2.1. Materials

Potassium 2,5-dihydroxybenzenesulfonate (SHQ), 2,6-difluorobenzonitrile (DFBN), deuterated dimethyl sulfoxide (DMSO-d6) and N,N-dimethyl formamide (DMF) were obtained from Sigma Aldrich (Shanghai, China). Bisphenol A (BPA), phenolphthalein (PP), zinc (Zn), sodium hydroxide (NaOH), potassium carbonate (K_2_CO_3_), N-methyl pyrrolidone (NMP), toluene, ethanol, tetrahydrofuran (THF), N,N-dimethylformamide (DMF), dichloromethane (CH_2_Cl_2_) and sodium dodecyl sulfate (SDS) were received from Chengdu Kelong Chemical Co. (Chengdu, China). Methylene blue (MB) and methyl orange (MO) were purchased from Sinopharm chemical reagent (Shanghai, China). Phenolphthalin (PPL) was synthesized from phenolphthalein (PP), Zn and NaOH.

### 2.2. The Synthesis of B-b-S-P

**Figure 1:**
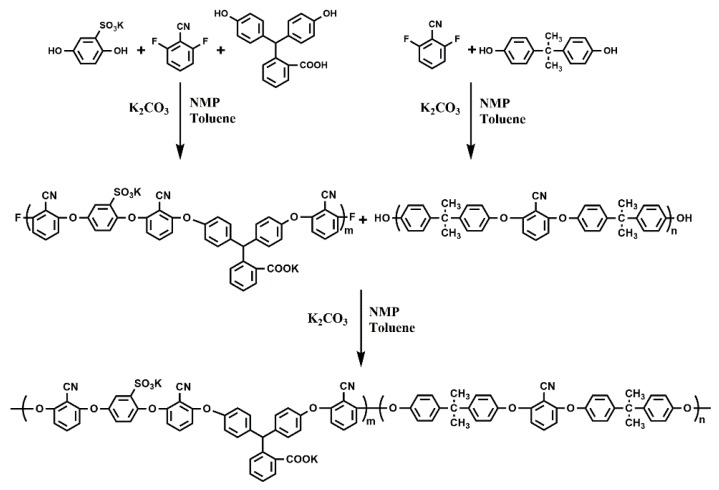
The synthesis route of amphiphilic block B-*b*-S-P.

The new amphiphilic block poly(arylene ether nitrile) (B-*b*-S-P) was synthesized on the basis of our previous work with a slight modification, and the specific synthesis route is displayed in Figure 1 [26]. With an excess ratio of reactants at 5%, the hydrophilic segment (*b*-S-P) was synthesized from SHQ (6.84 g, 30 mmol), PPL (9.549 g, 30 mmol) and DFBN (8.757 g, 63 mmol). Similarly, polymerization of hydrophobic segment (*b*-B) was conducted with BPA (14.382 g, 63 mmol) and DFBN (8.34 g, 60 mmol). Firstly, the hydrophilic and hydrophobic oligomers were obtained in two three-necked flasks, respectively. With the help of K_2_CO_3_ and toluene, the nucleophile in two flasks accomplished dehydration and pre-polymerization in 2–3 h. Then, the two received oligomers were uniformly mixed together for the ensuing reaction to proceed at 175 °C. Subsequently, the obtained polymer was precipitated in ethanol and further washed with diluted hydrochloric acid and an aqueous solution. Furthermore, the obtained product was immersed in NaOH solution to realize the deprotonation of B-*b*-S-P. Finally, the purified B-*b*-S-P was dried under vacuum at 80 °C for 48 h after extra NaOH was removed.

### 2.3. Preparation of the B-b-S-P Microspheres

A microemulsion method was adopted to prepare the polymeric microspheres according to our previous work with slight modifications [26]. In a typical process, 10 mL of aqueous solution containing 30 mg SDS was firstly prepared in a vial. Then, a mixture containing B-*b*-S-P (2 mg), CH_2_Cl_2_ (0.9 mL) and a variable amount of THF was added into above vial under vigorous stirring. Specifically, three different THF contents (0.1, 0.5 or 1 mL) were adjusted in the microemulsion system. After a continuous stirring for 12 h, the products were collected by centrifugation and purification using deionized water 3 times. In addition, the concentrations of components’ materials that were used in the preparation process were proportionally amplified by 50 times to investigate the structural stability of the B-*b*-S-P microspheres. Additionally, all of the microspheres that were applied in adsorption experiments were obtained from the amplified microemulsion system.

### 2.4. Batch-of-Dye Adsorption

Generally, adsorption experiments were conducted using 5 mg of B-*b*-S-P microspheres and 10 mL of MB solution within a vial, which was inhibited in a thermostat water bath with a magnetic stirrer. The adsorption experiments were performed under vigorous stirring with certain temperature and pH value. Moreover, 5 mg of B-*b*-S-P microspheres were added into a mixed dye solution containing 5 mL MB (20 mg L^−1^) and 5 mL MO (20 mg L^−1^) to evaluate the selective adsorption property of microspheres. In certain time intervals, the dye solutions were collected and then tested by UV-Vis spectrophotometer. On the basis of dyes’ concentration changes, the instantaneous adsorption capacities ($q_{t}$) and equilibrium adsorption capacities ($q_{e}$) of the microspheres were calculated by Equations (1) and (2) were displayed [28].

(1) $$q_{t}=\left(\frac{C_{o}-C_{t}}{m}\right)\times V$$

(2) $$q_{e}=\left(\frac{C_{o}-C_{e}}{m}\right)\times V$$

where $C_{o}$ (mg L^−1^) represents the initial concentration of the dye solution; $C_{e}$ (mg L^−1^) and $C_{t}$ (mg L^−1^) are the dye concentrations in solution at equilibrium time; and given time t. V (L) and *m* (mg) represent the volume of dye solution and the mass of adsorbent, respectively. 

### 2.5. Characterization

The characteristic functional groups of B-*b*-S-P were examined by Fourier transform infrared spectroscopy (Shimadzu 8400S FTIR spectrometer, Kyoto, Japan) and ^1^H unclear magnetic resonance spectrometry (Bruker AV II-400, Bruker, Switzerland, DMSO-d6, δ = 2.50 ppm). X-ray photoelectron spectroscopy (XPS) (Thermo Scientific Escalab 250Xi, Waltham, MA, USA) of B-*b*-S-P before and after the adsorption of MB was performed to expound on the changes of typical chemical bonds. The molecular weight and distribution were recorded by Waters Breeze 2 HPLC system (Waters corporation, Milford, CT, USA) with a gel permeation chromatography (GPC) method using DMF as the eluent and poly(methyl methacrylate) as the standard. (The weight average molecular weights (Mw) of B-*b*-S-P, the hydrophilic segment (*b*-S-P) and the hydrophobic segment (*b*-B) were 73817, 8943 and 8230 g mol^−1^, respectively.) Thermal gravimetric analysis (TGA) and derivative thermogravimetric analysis (DTG) of B-*b*-S-P were obtained by a TA Instruments of TGA-Q50 (Newcastle, DE, USA) at a heating rate of 20 °C min^−1^ under a nitrogen atmosphere. Additionally, the B-*b*-S-P was heated at a rate of 10 °C min^−1^ under a nitrogen atmosphere for differential scanning calorimetry (DSC) using a TA Instrument, DSC-Q100 (Newcastle, DE, USA). Scanning electron microscopy (SEM, JMS-6490LV, JEOL, Akishima, Japan) and transmission electron microscopy (TEM, JEM-2100F, JEOL, operating at 200 kV, Akishima, Japan) were employed to characterize the morphology of B-*b*-S-P microspheres. Ultraviolet-visible (UV-Vis) absorption spectra of MB in aqueous solutions were detected with a UV-Vis spectrophotometer (TU 1901, Persee, Beijing, China). The size distributions of microspheres were calculated by a statistical software called “Image J.”

## 3. Results and Discussion

### 3.1. Characterization of B-b-S-P

**Figure 2:**
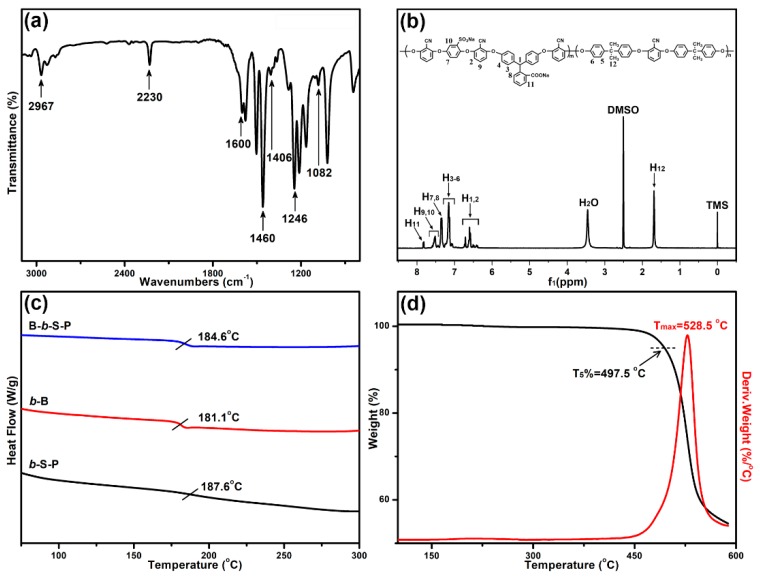
The FTIR (**a**), ^1^H NMR (**b**), differential scanning calorimetric (DSC) (**c**) and thermal gravimetric analysis (TGA) (**d**) spectra of synthesized B-*b*-S-P.

The chemical structure and thermal stability of B-*b*-S-P were both characterized. As shown in the FTIR spectrum in Figure 2a, the absorption bands at 2967 and 2230 cm^−1^ were attributed to the stretching vibration of C–H on methyl groups and the symmetric stretching vibration on nitrile groups, respectively. Owing to the deprotonation of B-*b*-S-P, the absorption band of carboxylate groups was found at 1406 cm^−1^. The characteristic bands belonging to skeleton vibrations of benzene rings were found at 1600 and 1460 cm^−1^. In addition, the peaks around 1246 and 1082 cm^−1^ were assigned to aromatic ether and sulfonate groups, respectively. With DMSO-d6 as the standard solvent, the ^1^H NMR spectra of B-*b*-S-P was detected and shown in Figure 2b. The peaks at 2.5 and 3.46 ppm were ascribed to DMSO-d6 and H_2_O, respectively. The primary hydrogen atoms of methyl groups were observed at 1.69 ppm, certifying the existence of a hydrophobic block containing BPA. Moreover, the characteristic peak assigned to the tertiary hydrogen atom on PPL was exhibited at 6.66 ppm. As for the peaks ranging from 6.73 to 7.83 ppm, they would be attributed to the hydrogen atoms on benzene rings. Figure 2c presented the DSC spectra of hydrophilic B-*b*-S-P, hydrophilic *b*-S-P and hydrophobic *b*-B, whose glass transition temperatures (*T*_g_) were about 184.6, 187.6 and 181.1 °C, respectively. Moreover, the 5% weight loss (*T*_5_%) temperature of B-*b*-S-P was at 497.5 °C and its maximum decomposition rate temperature (*T*_max_) was about 528.5 °C in the nitrogen atmosphere, as in the TGA and DTG curves shown in Figure 2d. These characterizations certificated that the amphiphilic block B-*b*-S-P was successfully synthesized, with high-temperature resistance, which should contribute to a wider application of B-*b*-S-P microspheres in harsh environments.

### 3.2. Preparation of the B-b-S-P Microspheres

**Figure 3:**
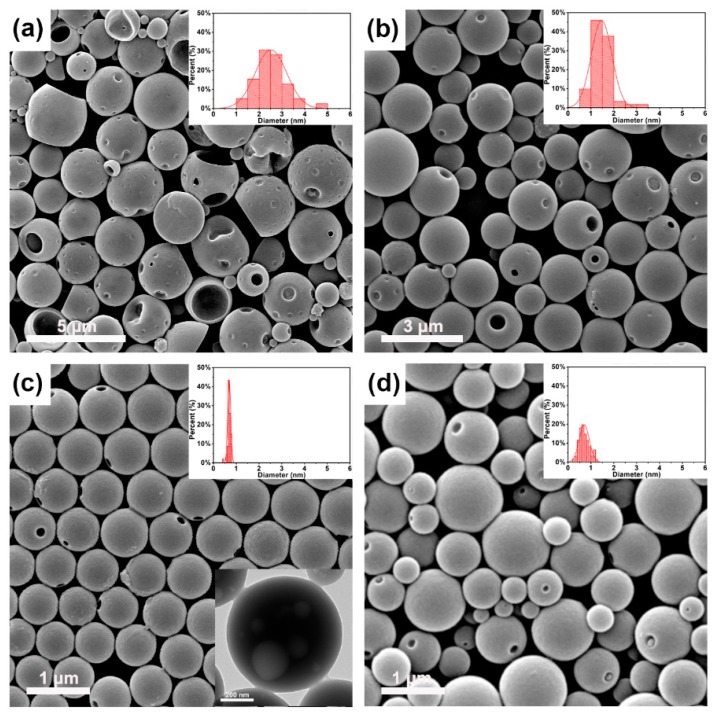
The SEM images and corresponding particle size distributions of B-*b*-S-P microspheres prepared with the assistance of 0.1 mL (**a**), 0.5 mL (**b**), and 1 mL (**c**) of THF and in the amplified oil-in-water (“O/W”) system (**d**). Insets: typical TEM image of B-*b*-S-P microspheres.

**Figure 4:**
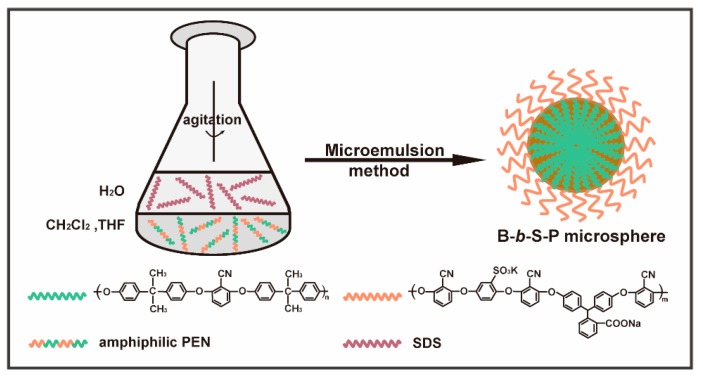
Schematic diagram of the preparation of B-*b*-S-P microspheres.

The morphologies and related particle size distributions of B-*b*-S-P aggregates that were prepared in an “oil in water” (O/W) microemulsion system were displayed in Figure 3. With the same range of horizontal and vertical coordinates, the particle size distributions of B-*b*-S-P microspheres from Figure 3a to Figure 3c obviously got more and more narrow. In the presence of 0.1 mL THF, the aggregates obtained in Figure 3a presented an irregular and fractured spherical structure with some pits, which also exhibited a wide particle size distribution and an average diameter ~2.5 μm. When THF content was increased to 0.5 mL, the relatively smaller microspheres with an average diameter of 1.5 μm were detected in Figure 3b. Besides, no more obvious cracked microspheres were observed with the exception of little pits. With the THF content continuously increased to 1 mL, the microspheres received were uniform in size with an average diameter of 0.7 μm, as the SEM image and particle size distribution of microspheres display in Figure 3c. Moreover, the TEM image shown further verified the integrity and roundness of microspheres. These SEM images indicated that the B-*b*-S-P was competent at preparing integrate microspheres; moreover, the THF content in the “O/W” system was crucial for preparing uniform B-*b*-S-P microspheres. Since the hydrophobic segment of *b*-B was soluble in THF, enough THF would be beneficial for the stretching of *b*-B, also leading to uniform and integrate microspheres. Meanwhile, insufficient THF might have impeded the extending of B-*b*-S-P chains, resulting in unregular assembly with cracked microspheres [17,20]. Furthermore, an “O/W” system referring to the amplified components’ proportions in Figure 3c was applied to prepare B-*b*-S-P microspheres, because a quantity of B-*b*-S-P microspheres were in need to evaluate their dye adsorption performance. It was found that the B-*b*-S-P microspheres obtained (Figure 3d) displayed a wide size distribution compared with the microspheres in Figure 3c, while no obvious cracks or pits were observed and the average diameter was also close to the result in Figure 3c. The relatively stable morphology and size distribution of B-*b*-S-P microspheres should contribute to a wider application in many fields. For example, the microspheres might act as supporter for loading a photocatalyst or encapsulate an active drug for multimodal imaging and drug delivery [29,30]. As shown in Figure 4, the microspheres were obtained on the basis of the selective solubility of amphiphilic block B-*b*-S-P in the “O/W” system with the assistance of SDS. It should be noted that the hydrophilic surfactant SDS in the “O/W” system acted as an emulsifier to reduce the interfacial tension and energy requirement, which was sufficient to enhance the stability of resulted microspheres.

### 3.3. Adsorption Kinetics

**Figure 5:**
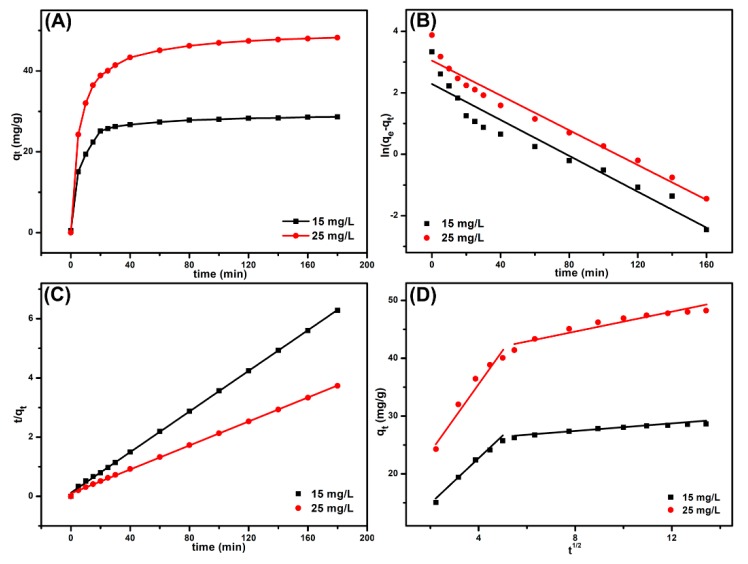
The effect of contact time and initial concentration on methylene blue (MB) adsorption capacity (**A**): pseudo-first-order model (**B**), pseudo-second-order model (**C**) and intraparticle diffusion model (**D**) for the adsorption of MB onto B-*b*-S-P microspheres.

Adsorption kinetic experiments were conducted at 25 °C in a neutral condition, and the concentrations of MB solutions were 15 and 25 mg L^−1^, respectively. As shown in Figure 5A, the adsorption capacity of B-*b*-S-P microspheres conspicuously increased at the initial stage, and then slowed down until it reached equilibrium. The fast adsorption would have been due to the fact that it was easy for MB molecules to occupy most of vacant surface sites on B-*b*-S-P microspheres during the initial stage, while the repulsive force between dyes and adsorbent might have restrained further adsorption of MB on remaining vacant surface sites [31]. Moreover, B-*b*-S-P microspheres presented a high equilibrium adsorption capacity to MB of 25 mg L^−1^, which was mainly attributed to that high initial MB concentration having supplied a driving force to relieve the mass transfer resistance of dyes. Herein, the pseudo-first-order (Figure 5B), pseudo-second-order (Figure 5C) and intraparticle diffusion (Figure 5D) models were simulated to analyze the adsorption isotherm, whose models were calculated with Equations (3)–(5) [32]:

(3) $$q_{t}=q_{e}\left(1-e^{-k_{1}t}\right)$$

(4) $$q_{t}=\frac{k_{2}q_{e}^{2}t}{1+k_{2}q_{e}t}$$

(5) $$q_{t}=k_{i}t^{0.5}+C$$

where *k*_1_ (min^−1^), *k*_2_ (g mg^−1^ min^−1^) and $k_{i}$ (mg g^−1^ min^−0.5^) are the rate constants of pseudo-first order, pseudo-second order and intraparticle diffusion model, respectively. *t* (min) is the contact time and C (mg g^−1^) is a constant related to adsorption steps. The corresponding spectra in Figure 5A,B indicated that the adsorption data were more fitted with the pseudo-second-order model than pseudo-first-order model. Moreover, the collected data in Table 1 exhibited that the linear correlation coefficient in pseudo-second order model was closer to 1 and the calculated *q_e_* (cal.) was also closer to the experimental *q_e_* (exp.). In addition, the simulated curves based on intraparticle diffusion model demonstrated that there were two steps in the dye diffusion process, as shown in Figure 5D [33]. The first adsorption step was known as the film diffusion stage, which referred to the diffusion of MB molecules from the solution to the surfaces of B-*b*-S-P microspheres. The subsequent adsorption step, called the intra-particle diffusion stage benefited from the rough surface of the microspheres. Therefore, both film diffusion and intra-particle diffusion promoted the adsorption of MB onto B-*b*-S-P microspheres. The calculated parameters in Table 1 suggested that the slope in intra-particle diffusion stage was lower than the one in film diffusion stage, demonstrating that the intraparticle diffusion stage was a gradual process. What is more, that the calculated curves did not pass the origin implied the intraparticle diffusion was not the rate-limiting step.

### 3.4. Adsorption Isotherm

**Figure 6:**
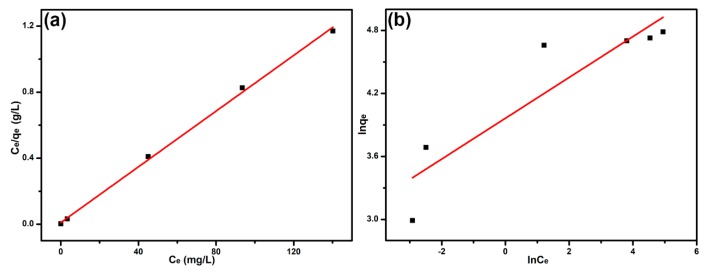
Langmuir isotherm (**a**) and Freundlich isotherm (**b**) for the adsorption of MB onto B-*b*-S-P microspheres.

The adsorption equilibrium isotherm was crucial to expound the adsorption behavior between adsorbate and adsorbent. A series of experiments were carried out using 10 mg B-*b*-S-P microspheres for the adsorption of 20 mL MB solutions with different concentrations (10–200 mg L^−1^). The typical Langmuir and Freundlich models were used to analyze the adsorption isotherm. It should be noted that the Langmuir model was suitable for analyzing the monolayer adsorption of homogeneous adsorbent, while the Freundlich isotherm assumed the adsorbent possessed a heterogeneous surface for multilayer adsorption. The two models were defined as the following equations [34]:

(6) $$\frac{C_{e}}{q_{e}}=\frac{1}{K_{L}q_{m}}+\frac{C_{e}}{q_{m}}$$

(7) $${lnq}_{e}={lnK}_{F}+\frac{1}{n}{lnC}_{e}$$

where $K_{L}$ (L mg^−1^) and *q_m_* (mg g^−1^) represent the Langmuir adsorption equilibrium constant and maximum adsorption capacity, respectively. $K_{F}$ and *n* are Freundlich constants. The Langmuir isotherm and Freundlich isotherm are exhibited in Figure 6a,b respectively. Relevant parameters calculated from the two models are collected in Table 2. Obviously, the Langmuir isotherm exhibited good linearity with a correlation coefficient of 0.9981, while the correlation coefficient in Freundlich isotherm was as low as 0.7975. Besides, the calculated adsorption capacity (119.05 mg g^−1^) was much closer to the experimental capacity (119.84 mg g^−1^) from the Langmuir isotherm, which suggested the adsorption sites at B-*b*-S-P microspheres were homogeneous and the adsorption followed a monolayer adsorption. Furthermore, Table 3 listed the maximum adsorption capacities of various polymer-derived adsorbents for MB, which indicated that B-*b*-S-P microspheres were more efficient than other adsorbents. It was also believed that enhanced adsorption performance of B-*b*-S-P microspheres would be achievable after suitable modifications.

Furthermore, a separation factor (*R_L_*) derived from the Langmuir isotherm was applied to evaluate the feasibility of the adsorption process, defined below [35]:

(8) $$R_{L}=\frac{1}{1+K_{L}C_{o}}$$

In general, the isotherms were classified as irreversible (*R_L_* = 0), favorable (0 < *R_L_* < 1), linear (*R_L_* = 1) and unfavorable (*R_L_* > 1). The *R_L_* in this work was calculated in the range of 0.0057–0.1035, suggesting the adsorption of MB onto B-*b*-S-P microspheres was favorable.

### 3.5. Adsorption Thermodynamics

**Figure 7:**
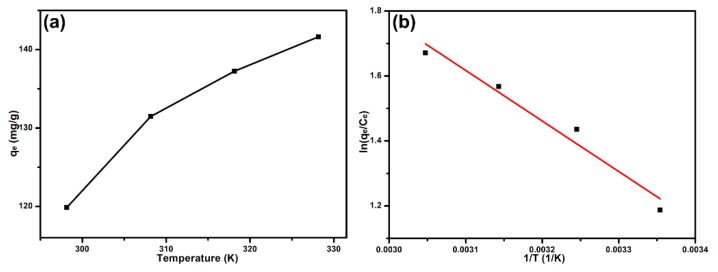
The effect of temperature on the adsorption of MB onto B-*b*-S-P microspheres (**a**) and the corresponding thermodynamic analysis (**b**).

Temperature was an important factor for dye’s adsorption. Figure 7a displays the varied equilibrium adsorption capacities (*q_e_*) of B-*b*-S-P microspheres for MB under four different adsorption temperatures (298–328K). The *q_e_* presented an obvious increasing trend and reached 141.62 mg g^−1^ at 328 K, suggesting that a higher temperature was beneficial for the adsorption of MB onto B-*b*-S-P microspheres. On the basis of Figure 7a, related thermodynamic parameters were calculated using following, Equations (9) and (10) [35]:

(9) $$\Delta G^{O}=-RT\ln K_{C}$$

(10) $$\ln K_{C}=-\frac{\Delta H^{O}}{RT}+\frac{\Delta S^{O}}{R}$$

where $\Delta G^{O}$ (kJ mol^−1^), $\Delta H^{O}$ (kJ mol^−1^) and $\Delta S^{O}$ (J mol^−1^ K^−1^) represent the changes of Gibbs free energy, enthalpy and entropy, respectively. $K_{C}$ (L g^−1^) is equal to the ratio of *q_e_* (mg g^−1^) and *C_e_* (mg L^−1^), R (8.314 J mol^−1^ K^−1^) is the universal gas constant and T (K) means the experimental temperature. From Van’t Hoff plot in Figure 7b, corresponding thermodynamic parameters were calculated and displayed in Table 4. It was found that the $\Delta G^{O}$ was not only negative but also demonstrated a decreasing trend along with the increased temperature, indicating the adsorption of MB onto B-*b*-S-P microspheres was spontaneous and especially favored at higher temperatures. The effect of temperature was also certificated from the positive $\Delta H^{O}$ of 1.2936 kJ mol^−1^, which implied that the adsorption of MB was endothermic. Moreover, the positive $\Delta S^{O}$ manifested showed that the adsorption of MB brought in an increased randomness among MB and B-*b*-S-P microspheres. Thus, it was believed that B-*b*-S-P microspheres were qualified and efficient at removing MB.

### 3.6. The Effect of Solution pH and Selective Adsorption for MB

**Figure 8:**
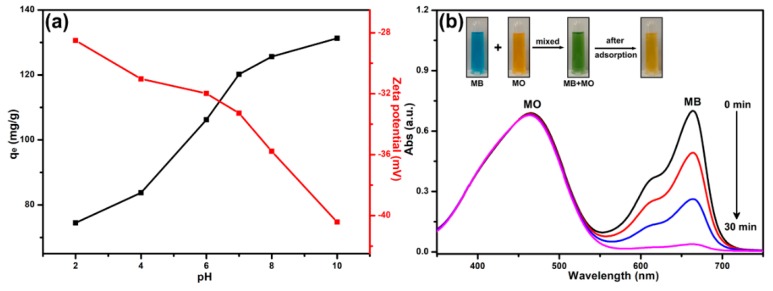
The effect of pH value on the adsorption capacity and zeta potential of B-*b*-S-P microspheres (**a**); the UV-Vis absorption variation curves of mixed dyes containing MB and MO in the presence of B-*b*-S-P microspheres (**b**). Inset: the color changes of mixed-dye solution after adsorption.

At different initial solution pH, the adsorption capacities and zeta potentials of B-*b*-S-P microspheres were investigated at 25 °C. As shown in Figure 8a, the adsorption capacities of B-*b*-S-P microspheres exhibited an obvious increase from 74 to 131 mg g^−1^ along with the pH value’s increase from 2 to 10, indicating that a basic solution was beneficial for the adsorption of MB onto B-*b*-S-P microspheres. In contrast, the zeta potentials were also detected to analyze the surface charge of B-*b*-S-P microspheres, which displayed a decreasing trend with the increasing solution-pH. Moreover, it should be noted that the zeta potential of B-*b*-S-P microspheres was maintained as a negative, which should be ascribed to the intrinsic deprotonation of carboxylate and sulfonate groups [28,40]. At the initial solution’s pH of 2, the low adsorption capacity was caused by the limited deprotonation of functional groups on B-*b*-S-P microspheres, which impeded their electrostatic interaction with cationic dye MB. When the deprotonation of functional groups was encouraged in an alkaline environment, the enhanced electrostatic interaction brought in excellent adsorption capacity of MB onto B-*b*-S-P microspheres [41]. Furthermore, the dye-mixture solution, simultaneously containing cationic MB and anionic MO, was prepared for further exploration of the adsorption property of B-*b*-S-P microspheres. As in the spectra shown in Figure 8b, the mixed solution before adsorption displayed two characteristic absorption bands at 664 and 464 nm, which were ascribed to MB and MO, respectively. After B-*b*-S-P microspheres were added into the dye-mixture, the peak of MB gradually weakened, while the peak of MO remained unchanged. The inset in Figure 8b exhibits the color change of the mixture solution from turquoise to orange yellow, suggesting the selective adsorption of B-*b*-S-P microspheres to cationic MB. Given that B-*b*-S-P microspheres were negatively charged, the maintained absorption band of MO could be attributed to the repulsive force between B-*b*-S-P microspheres and MO. In conclusion, electrostatic interaction was considered to be the main force contributing the dye-adsorption of B-*b*-S-P microspheres.

### 3.7. Adsorption Mechanism

**Figure 9:**
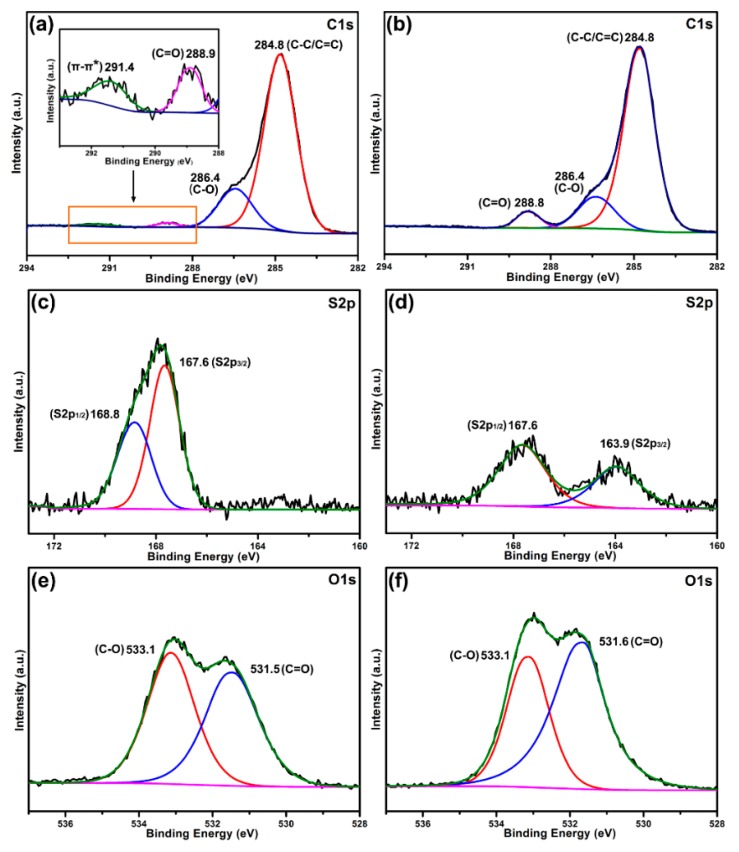
Peak-fitting X-ray photoelectron spectroscopy (XPS) spectra in the C1s, S2p and O1s of B-*b*-S-P microspheres before and after MB adsorption: C1s of B-*b*-S-P (**a**) and B-*b*-S-P/MB (**b**); S2p of B-*b*-S-P (**c**) and B-*b*-S-P/MB (**d**); O1s of B-*b*-S-P (**e**) and B-*b*-S-P/MB (**f**).

**Figure 10:**
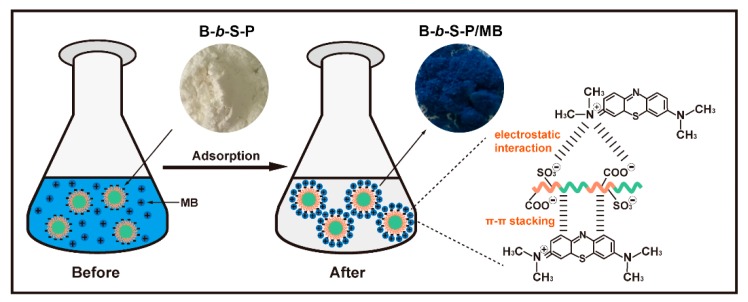
Schematic illustration and supposed mechanism of the adsorption of MB onto B-*b*-S-P microspheres.

To gain an insight into the adsorption mechanism, X-ray photoelectron spectroscopy (XPS) of B-*b*-S-P microspheres before (Figure 9a,c,e) and after (Figure 9b,d,f) adsorption of MB were contrasted. The C1s in the spectra of B-*b*-S-P microspheres (Figure 9a) were fitted onto four peaks, which corresponded to C–C (284.8 eV), C–O (286.4 eV), C=O (288.9 eV) and π–π* satellite (291.4 eV) peaks. Specifically, the satellite peak at 291.4 eV was derived from the π–π* transition in aromatic ring, whereas the π–π* satellite peak was not detected after adsorption, implying the π–π stacking interaction might have promoted the adsorption of MB onto B-*b*-S-P microspheres, as per the result shown in Figure 9b. As for the S2p spectra in Figure 9c, two peaks at 167.6 eV (S2p_3/2_) and 168.8 eV (S2p_1/2_) belonging to sulfonate group of B-*b*-S-P microspheres were obtained. After the adsorption of MB, the S2p spectra of B-*b*-S-P microspheres presented two clear split peaks with decreased intensities. As shown in Figure 9d, the splitting peaks at 163.9 eV (S2p_3/2_) and 167.6 eV (S2p_1/2_) should be attributed to the sulfur of the phenothiazine structure in MB, indicating the successful adsorption of MB onto B-*b*-S-P microspheres. What is more, the O1s in the spectrum of B-*b*-S-P microspheres exhibited two peaks at 531.5 eV (C=O) and 533.1 eV (C–O), as shown in Figure 9e. Due to the oxygen atom having a priority to accept an electron, the intensity of O1s peaks slightly increased after the adsorption of MB, suggesting the electrostatic interaction between them [42,43]. As a result, the adsorption of MB onto B-*b*-S-P microspheres would be mainly dependent on the electrostatic interaction and π–π stacking interaction. The possible adsorption process and mechanism are illustrated in Figure 10; the white and blue powders are the B-*b*-S-P microspheres before and after the adsorption of MB, respectively.

## 4. Conclusions

In summary, a kind of newly synthesized amphiphilic block poly(arylene ether nitrile) was successfully applied to preparing uniform B-*b*-S-P microspheres, which displayed excellent adsorption capacity for cationic dye MB. The adsorption kinetics of MB onto B-*b*-S-P microspheres followed the pseudo-second-order model and the intraparticle diffusion model, indicating the intraparticle diffusion was not the rate-limiting step. Moreover, the Langmuir isotherm was more suitable to explain the homogeneous adsorption sites on the surfaces of B-*b*-S-P microspheres. The experimental maximum adsorption capacity of B-*b*-S-P microspheres was calculated to be 119.84 mg g^−1^ at 25 °C in neutral conditions, and B-*b*-S-P microspheres were certified to be capable of selectively removing cationic MB, while MO remained unchanged in the mixed-dye solution. In addition, alkaline conditions and a higher temperature were beneficial for removing MB. Removal benefited from the functional groups and conjugated structure of B-*b*-S-P microspheres, both electrostatic interactions and the π–π stacking interaction promoted the adsorption of MB. It is expected that the B-*b*-S-P microspheres have great potential as nanoreactors to exert their dye-disposing specialty.

## Figures and Tables

**Table 1:** The kinetic parameters of adsorption of MB onto B-*b*-S-P microspheres.

Models	Parameters	15 mg L^−1^	25 mg L^−1^
Pseudo-first-order	*k*_1_ (min^−1^)	0.0292	0.0283
	*q_e_* (cal.) (mg g^−1^)	9.8384	21.066
	*q_e_* (exp.) (mg g^−1^)	28.643	48.243
	*R* ^2^	0.9163	0.9587
Pseudo-second-order	*k*_2_ (g mg^−1^ min^−1^)	0.0120	0.0042
	*q_e_* (cal.) (mg g^−1^)	29.155	49.505
	*q_e_* (exp.) (mg g^−1^)	28.643	48.243
	*R* ^2^	0.9996	0.9994
Intraparticle diffusion	*k_i_*_1_ (mg g^−1^ min^−0.5^)	3.8658	5.7591
	*C*_1_	6.8537	12.744
	*R*_1_^2^	0.9843	0.9413
	*k_i_*_2_ (mg g^−1^ min^−0.5^)	0.2945	0.8029
	*C*_2_	24.930	38.259
	*R*_2_^2^	0.9395	0.8994

**Table 2:** Adsorption isotherm constants for the adsorption of MB onto B-*b*-S-P microspheres.

Isotherms	Parameters	Temperatures (K) 298.15
Langmuir	*q_m_* (mg g^−1^)	119.05
	*K_L_* (L mg^−1^)	0.8660
	*R* ^2^	0.9981
Freundlich	*K_F_* (L mg^−1^)	52.657
	*n* ^−1^	0.1943
	*R* ^2^	0.7975

**Table 3:** Comparation of maximum adsorption compacity (*q_m_*) of MB by various polymeric adsorbents.

Adsorbent	*q_m_* (mg g^−1^)	Reference
DPA microspheres	90.7	[14]
CS-MPONs nanocomposites	104	[15]
PCPP microspheres	50.7	[35]
PZS nanospheres	20.0	[36]
Polyamide-vermiculite nanocomposites	76.42	[37]
Poly(methacrylate)/silica hybrid materials	91.324	[38]
PDA-rGO-kaolin composite	39.663	[39]
B-*b*-S-P microspheres	119.84	This work

**Table 4:** Thermodynamic parameters of the adsorption of MB onto B-*b*-S-P microspheres.

*T* (K)	Thermodynamic Parameters			
	ln*K*	Δ*G^O^* (kJ mol^−1^)	Δ*S^O^* (J mol^−1^ K^−1^)	Δ*H^O^* (kJ mol^−1^)
298	1.1873	−2.9431	53.545	1.2936
308	1.4360	−3.6790	—	—
318	1.5672	−4.1454	—	—
328	1.6709	−4.5585	—	—
